# The Diagnostic Value of Coagulation Indicators and Inflammatory Markers in Distinguishing Between Strangulated and Simple Intestinal Obstruction

**DOI:** 10.1097/SLE.0000000000000982

**Published:** 2021-07-21

**Authors:** Haijun Li, Dian Sun, Duocheng Sun, Zhong Xiao, Jiongyu Zhuang, Chunlei Yuan

**Affiliations:** * Prenatal Diagnosis Center; Departments of † Emergency; ‡ Radiological; § Clinical Laboratory, Boai Hospital of Zhongshan Affiliated to Southern Medical University, Zhongshan, Guangdong, China

**Keywords:** intestinal obstruction, strangulated intestinal obstruction, simple intestinal obstruction, fibrinogen, C-reactive protein

## Abstract

Intestinal obstruction with strangulation can be life-threating, and it is critical to make an accurate and timely diagnosis for emergency surgery.

**Objective:**

This was aimed to investigate the value of coagulation indicators and inflammatory markers in distinguishing between strangulated and simple intestinal obstruction.

**Materials and Methods:**

Fifty-four patients with intestinal obstruction were retrospectively studied. The correlation between coagulation indicators and inflammatory markers with intestinal obstruction was analyzed. Receiver operating characteristic curves were created to assess their ability in discriminative diagnosis.

**Results:**

Levels of fibrinogen (Fib), C-reactive protein (CRP), neutrophil ratio, and D-Dimer were significantly greater, while thrombin time was significantly shorter in strangulated intestinal obstruction compared with simple intestinal obstruction. Furthermore, Fib levels in the necrosis subgroup of strangulated intestinal obstruction were significantly higher than those in the ischemia subgroup and simple intestinal obstruction group. The areas under the receiver operating characteristic curve were 0.58 for white blood cells, 0.78 for CRP, and 0.80 for Fib. Using the optimal cutoff values of Fib (3.71 g/L) and CRP (14.54 mg/L), the sensitivity, specificity, positive predictive value, and negative predictive value in discriminating between strangulated intestinal obstruction and simple intestinal obstruction were 51.43%, 100%, 100%, and 52.78% for Fib, and 56.25%, 94.44%, 94.74%, and 54.84% for CRP, respectively.

**Conclusions:**

Fib and CRP demonstrate good performance in predicting strangulation and are indicative of intestinal necrosis and ischemia. The combination of this coagulation indicator and inflammatory marker holds potential for better discrimination between strangulated and simple intestinal obstruction.

Intestinal obstruction, also known as small bowel obstruction, is a common clinical condition requiring gastrointestinal surgery; it is generally classified into the following 2 types: simple intestinal obstruction and strangulated small intestinal obstruction.^1^,^2^ Of these, strangulated small intestinal obstruction is a more serious condition with a significantly higher mortality rate than simple intestinal obstruction.^1^,^2^ In fact, if left untreated or treatment is delayed, strangulated intestinal obstruction can be life-threatening and is ultimately fatal, with a mortality rate of up to 30%.^1^,^2^ Therefore, an accurate diagnosis and timely implementation of appropriate surgical treatment are critically important for good clinical outcomes of patients with strangulated intestinal obstruction. Currently, computed tomography is widely used in clinical practice for the preoperative diagnosis and assessment of strangulated intestinal obstruction-associated complications. However, this diagnostic modality has a number of flaws. For instance, the use of computed tomography exposes the patients to ionizing radiation, which can increase the risk of developing radiation-associated cancer. In addition, it remains difficult to differentiate strangulated intestinal obstruction from simple intestinal obstruction. Therefore, there is an urgent need to develop a better diagnostic method to distinguish between simple intestinal obstruction and strangulated intestinal obstruction, the latter of which requires an emergency surgical intervention.

Coagulation indicators, particularly fibrinogen (Fib), have been reported previously to be useful markers in the diagnosis of coagulation disorders that may result from ischemia and hypoxia.^3^ Schoots et al^4^ have reported intraluminal coagulation and fibrin deposition in a mouse model of intestinal ischemia-reperfusion. In another study using an acute strangulation hernia model in rats, Zeybek and colleagues showed that the levels of D-Dimer (D-D) increased as the ischemia worsened, and the alteration was significantly correlated with intestinal necrosis. In addition, the same study found that the white blood cell (WBC) counts were significantly higher in the experimental group with intestinal necrosis compared with the control group. Strangulated intestinal obstruction is pathologically accompanied with defects in intestinal microcirculation, microthrombosis, blood clotting, hypoxia, tissue ischemia, and even intestinal necrosis. Thus, we hypothesized that the main pathologic changes may lead to alterations in the levels of coagulation indicators, including Fib. To date, the use of these coagulation indicators, especially Fib, for the differential diagnosis of strangulated intestinal obstruction versus simple intestinal obstruction has not been investigated in depth.

In this retrospective study of patients with intestinal obstruction, we aimed to determine whether an association between coagulation indicators as well as inflammatory markers and the 2 types of intestinal obstruction exists. In addition, we assessed their diagnostic value in differentiating strangulated and simple intestinal obstruction.

## MATERIALS AND METHODS

### Study Subjects

All patients with intestinal obstruction who underwent surgical treatment in our hospital from January 2013 to November 2017 were retrospectively studied. All patients who had coagulation disorders or special physiological conditions after taking anticoagulant drugs were excluded. Patients meeting inclusion criteria for: (1) all cases were hospitalized patients with intestinal obstruction; (2) all cases were surgical patients with intestinal obstruction; (3) all cases were confirmed by operation and detailed intraoperative records and/or pathologic confirmation; (4) all cases met the diagnostic criteria of intestinal obstruction in the seventh edition of Huang Jiasi surgery. And exclusion criteria for: (1) diseases affecting coagulation function, such as coagulation diseases, leukemia, and advanced malignant tumor; (2) special physiological conditions, such as middle and late pregnancy and people over 80 years old; (3) people who have used anticoagulants; (4) dynamic and hemodynamic ileus. All the patients hemodynamically stable at time of operation. A total of 54 intestinal obstruction patients, including 21 cases of adhesive intestinal obstruction, 11 cases with intestinal obstruction due to external hernia, 10 cases with intestinal torsion, 5 cases with intestinal obstruction in the megacolon because of long term intestinal feces, 5 cases with intestinal obstruction due to fecalith, and 2 cases with intestinal obstruction due to a benign tumor, were retrospectively enrolled in this study. According to the intraoperative records, postoperative diagnosis, and pathologic results, the patients were divided into the simple intestinal obstruction group (n=19) or the strangulated intestinal obstruction group (n=35), which consisted of 16 cases of ischemia and 19 cases of necrosis. All patients were tested for coagulation indicators, C-reactive protein (CRP), and whole blood count.

The study protocol was reviewed and approved by the Research Ethics Committee at the Boai Hospital of Zhongshan affiliated to Southern Medical University.

### Statistical Analysis

Statistical analysis was performed using SPSS 22.0, with the values shown as mean±SD. We performed descriptive analysis, univariate analysis, and correlation analysis for any 2 groups using the Student *t* test, the Mann-Whitney *U* test, and Pearson Correlation Analysis test, respectively. We conducted analysis of variance or the Kruskal-Wallis test among the groups. *P*<0.05 was considered statistically significant. The diagnostic value of routine coagulation indicators for strangulated intestinal obstruction was determined using receiver operating characteristic curve (ROC) analysis.

## RESULTS

### Characteristics of the Study Subjects

A total of 94 patients underwent gastrointestinal surgery for suspicion of intestinal obstruction, 54 of whom were finally included and retrospectively studied. Forty patients were excluded from this study due to the following conditions: intestinal cancer (32), urinary tract cancer (1), cerebral infarction (1), tuberculosis (1), late pregnancy (1), heparin treatment (1), and aged more than 80 years old (3). The characteristics of the study subjects are summarized in Table 1. There were 35 males and 19 females, with a mean age of 15.00±23.43 years, ranging from 6 months to 79 years. The mean time between the diagnosis of intestinal obstruction and the blood examinations was 76±80 hours, ranging from 4 to 360 hours. According to the type of intestinal obstruction, the study subjects were allocated into 2 groups: simple intestinal obstruction group (simple group, n=19), and strangulated intestinal group (strangulated group, n=35). There were no significant differences in terms of sex or age between the 2 groups. Of note, there was a significant difference in the time between disease diagnosis to blood extraction between the 2 groups (*P*<0.05) due to the fact that the majority of cases in the simple group were treated with gastrointestinal surgery following a long period of time after conservative treatment was ineffective. And all the patients were hemodynamically stable at time of operation. As simple intestinal obstruction did not worsen, it did not affect the statistical analysis.

**TABLE 1:** Comparison of Preoperative Test Results (Mean±SD) Between Simple and Strangulated Intestinal Obstruction

	Simple Intestinal Obstruction	Strangulated Intestinal Obstruction	*P*
WBC (×10^9^)	10.69±4.58	13.44±7.74	0.162
NEUT (%)	56.92±23.07	69.95±17.31	0.023
PLT (×10^9^)	412.95±172.26	388.40±179.09	0.628
CRP (mg/L)	6.19±5.48	44.58±58.63	0.008
PT (s)	14.12±1.24	14.82±2.16	0.196
APTT (s)	40.35±5.61	39.26±5.99	0.516
Fib (g/L)	2.79±0.67	3.88±1.25	0.001
TT (s)	17.32±1.18	16.35±1.38	0.012
D-D (μg/mL)	0.63±0.44	3.14±2.42	0.036

APTT indicates activated partial thromboplastin time; CRP, C-reactive protein; D-D, D-dimer; Fib, fibrinogen; NEUT, neutrophil ratio; PLT, platelet count; PT, prothrombin time; TT, thrombin time; WBC, white blood cell count.

### **C**omparative Analysis of the Preoperative Blood Examination Results Between the Simple and Strangulated Groups

The results of the preoperative blood examinations, including WBC, neutrophil ratio (NEUT), platelet count, CRP, prothrombin time (PT), activated partial thromboplastin time (APTT), Fib, thrombin time (TT), and D-dimer (DD), were compared between the simple intestinal obstruction group and the strangulated intestinal obstruction group. As shown in Table 1, the values of Fib, CRP, NEUT, and D-D were significantly greater in the strangulated intestinal obstruction group than in the simple intestinal obstruction group (*P*<0.01). In addition, the TT values were significantly lower in the strangulated intestinal obstruction group than in the simple intestinal obstruction group (*P*<0.05).

To examine the potential correlation between the coagulation indicators and the severity of the strangulated intestinal obstruction, the patients in the strangulated group were further divided into 2 subgroups: ischemia subgroup and necrosis subgroup, and the values of WBC, NEUT, platelet count, CRP, PT, APTT, Fib, TT, and D-D were compared. We found that the Fib level in the necrosis subgroup was significantly higher than that in the simple intestinal obstruction group and the ischemia subgroup (*P*<0.01 and <0.05, respectively). The values of CRP between in the simple intestinal obstruction group and the necrosis subgroup as well as between the ischemia subgroup and the necrosis subgroup were also statistically different (*P*<0.01). In addition, the values of NEUT, PT, and D-D between the simple group and the necrosis subgroup were significantly different (*P*<0.05). Finally, TT was significantly shorter in the simple intestinal obstruction group compared with the necrosis subgroup (*P*<0.01). Due to the relatively small number of cases with D-D examination results, no further analysis was made.

### **P**erformance of the Coagulation Indicators for Discriminating Between Strangulated Intestinal Obstruction and Simple Intestinal Obstruction

**FIGURE 1:**
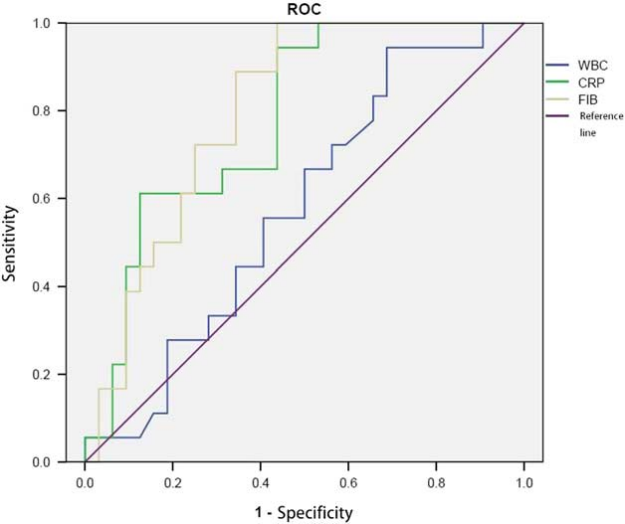
Receiver operating characteristic (ROC) curves for white blood cell count (WBC), C-reactive protein (CRP), and fibrinogen (Fib). The diagnostic ability of routine coagulation indicators for strangulated intestinal obstruction was determined using ROC analysis. *P*<0.05 was considered statistically significant.

ROC curve analysis was conducted to determine the performance of the coagulation indicators for discriminating between strangulated intestinal obstruction and simple intestinal obstruction. As shown in Figure 1, the areas under the ROC curve (AUCs) were 0.58 for WBC, 0.78 for CRP, and 0.80 for Fib. The optimal cutoff values of Fib and CRP for the differential diagnosis of strangulated intestinal obstruction were calculated as presented in Table 2. Using the cutoff values, the sensitivity, specificity, positive predictive value, and negative predictive value for Fib and CRP in discriminating between strangulated intestinal obstruction and simple intestinal obstruction were determined (Table 2).

**TABLE 2:** Optimal Cutoff Values, Sensitivity, Specificity, Positive Predictive Value, and Negative Predictive Value of Fib and CRP for the Diagnosis of Strangulated Intestinal Obstruction

	The Optimal Cutoff Point	Sensitivity (%)	Specificity (%)	Positive Predictive Value (%)	Negative Predictive Value (%)
Fib (g/L)	3.71	51.43	100	100	52.78
CRP (mg/L)	14.54	56.25	94.44	94.74	54.84

CRP indicates C-reactive protein; Fib, fibrinogen.

Correlation analysis of Fib, CRP, NEUT, and TT between the simple intestinal obstruction group and the strangulated intestinal obstruction group showed that Fib and CRP were significantly correlated with all forms of intestinal obstruction (*r*=0.63, *P*<0.01). Moreover, the values of Fib and CRP were negatively correlated with the TT (*r*=−0.54, *P*<0.01). Furthermore, Fib also had a positive correlation with CRP but a negative correlation with TT in the simple and the strangulated groups (Table 3).

**TABLE 3:** Analysis of Correlation Between Fib and CRP, NEUT, and TT

	CRP		NEUT		TT	
	*r*	*P*	*r*	*P*	*r*	*P*
Simple group Fib	0.56	<0.05	0.22	>0.05	−0.63	<0.01
Strangulated group Fib	0.58	<0.01	0.25	>0.05	−0.44	<0.01
Total intestinal obstruction Fib	0.63	<0.01	0.33	<0.05	−0.54	<0.01

CRP indicates C-reactive protein; Fib, fibrinogen; NEUT, neutrophil ratio; TT, thrombin time.

## DISCUSSION

This retrospective study of patients with simple or strangulated intestinal obstruction has the following major findings: (1) Fib, CRP, NEUT, and D-D were significantly greater in the patients with strangulated intestinal obstruction compared with those with simple intestinal obstruction; (2) TT was significantly lower in the strangulated intestinal obstruction group than in the simple intestinal obstruction group; (3) The Fib level was positively correlated with the severity of intestinal obstruction, with the highest values in the group with strangulated intestinal obstruction and necrosis; (4) The sensitivity, specificity, positive predictive value, and negative predictive value were 51.43%, 100%, 100%, and 52.78%, respectively, for Fib at the optimal cutoff point; while those values were 56.25%, 94.44%, 94.74%, and 54.84% for CRP at the optimal cutoff point, respectively, in discriminating strangulated intestinal obstruction from simple intestinal obstruction.

Strangulated intestinal obstruction is a serious clinical condition, and it requires a timely diagnosis and prompt surgical treatment. Extensive previous efforts have been made to search for diagnostic methodology to be used for patients with strangulation. The following pathologic changes can occur during the course of intestinal obstruction: dehydration, electrolyte disorders, secondary infection, intestinal microcirculation, blood clotting, microthrombosis, blood disorders, tissue ischemia, hypoxia, and ultimately necrosis; some of these effects (eg, tissue ischemia and hypoxia) can cause coagulation disorders. Thus, the coagulation tests were expected to indirectly reflect the status of the tissue blood flow. Schoots et al^4^ showed intraluminal coagulation and fibrin deposition in mouse intestinal ischemia-reperfusion experiments. In addition, in a rat acute strangulation hernia model, Zeybek et al^5^ demonstrated that the D-D level increased as the ischemic time progressed, compared with the control group, and the difference was statistically significant at 2 hours (*P*=0.027) and was related to intestinal necrosis. Moreover, the WBC was significantly increased at 6 hours compared with the control group (*P*=0.015). Altogether, these findings indicate that the serum D-D level is more valuable than the WBC in the diagnosis of early intestinal ischemia. Altinyollar et al^6^ also detected the D-D level by ligation of the superior mesenteric artery in rats; their results revealed that the plasma D-D level may be a useful indicator for an early diagnosis of acute mesenteric ischemia and intestinal necrosis.

Of a number of coagulation indicators, including PT, APTT, Fib, TT, and D-D, Fib can be used as a screening or diagnostic indicator of hypercoagulability, and it is also one of the molecular markers for monitoring coagulation, fibrinolysis, and thrombosis. During coagulation, Fib is eventually degraded into D-D with the help of thrombin and plasmin. However, the plasma D-D index is related to the degree of intestinal lesions and necrosis in the intestinal obstruction when the intestinal obstruction occurred. Yang et al^7^ studied 274 patients who had undergone surgery for their acute intestinal obstruction; the optimal cutoff value of the plasma D-D level was determined to be 1.965 mg/L, with a sensitivity of 84.0%, a specificity of 45.6%, a positive predictive value of 60.7%, and a negative predictive value of 74.0%. Moreover, when the cutoff value was 1.65 mg/L, reversible intestinal ischemia and intestinal necrosis could be distinguished. When the D-D level was combined with peritoneal irritation, there was a reliable negative predictive value, which helped to rule out intestinal necrosis. Similarly, Chen and Hu^8^ found that acute intestinal obstruction is usually accompanied by increased plasma D-D levels. Some patients with acute intestinal obstruction had no typical symptoms or signs, but intestinal necrosis was eventually found because the D-D level was determined to be gradually increasing in the plasma during the conservative treatment. So, Chen and Hu suggested that D-D might be an independent prognostic factor for intestinal necrosis of acute ileus and intestinal necrosis in the plasma. If continuous monitoring of the D-D level in the plasma is negative, then intestinal necrosis can be ruled out. Furthermore, Icoz et al^9^ suggested that D-D levels are significantly increased in ischemic small intestinal lesions, with a high sensitivity and a low specificity. In another study,^10^ 84 patients with intestinal obstruction were divided into 2 groups, including a simple intestinal obstruction group and a strangulated intestinal obstruction group. Extensive studies have demonstrated that the plasma D-D levels may help to detect or monitor acute mesenteric ischemia or intestinal necrosis.^10^–^15^ The results showed that D-D and other indicators not only can provide the basis for an early diagnosis of intestinal strangulation, but they also can be used to determine the degree of intestinal strangulation injury and intestinal necrosis. Therefore, the detection of coagulation indicators, especially D-D and Fib, is expected to indirectly reflect the pathologic changes of the intestinal lumen during intestinal obstruction as well as pathologic changes such as microcirculation disorders and microthrombus formation.

In addition, Chen and Hu^8^ analyzed a number of other scholars’ studies and suggested that D-D in plasma may be helpful for detecting or monitoring acute mesenteric ischemia or intestinal necrosis in patients with an acute abdomen. Zhong et al^16^ believed that D-D was of great value in the diagnosis of acute mesenteric ischemia. Most scholars^17^–^21^ consider that D-D has a high sensitivity and a low specificity and can be used as a screening and exclusion reference for acute mesenteric ischemia and intestinal necrosis. In another study, Lv et al^22^ retrospectively analyzed 19 patients with superior mesenteric vein thrombosis and showed that the D-D level in serum of the intestinal necrosis subgroup was significantly higher than that of the intestinal congestion group (*P*<0.05). The sensitivity, specificity, positive predictive value, and negative predictive value of the disease for intestinal necrosis were 70%, 89%, 87.5%, and 73%, respectively. Likewise, Lv and colleagues demonstrated that the concentration of D-D has an important predictive value for intestinal necrosis. Some other studies^11^–^13^,^23^–^26^ also determined that Fib, D-D, and other indicators of blood clots can ascertain the degree of appendix lesions in patients with appendicitis, especially for gangrene and perforation appendicitis. This evidence indirectly proves that it is feasible to detect the extent of intestinal lesions and enteric necrosis indirectly by measuring coagulation indices such as D-D and Fib, which can help to increase the accuracy of intestinal obstruction diagnosis. It also helps to guide clinicians to provide a reference for hospitalization and surgical exploration as early as possible.

### Limitations

In our study, TT differed not only between the simple and strangulation groups but also among the subgroups, including simple, strangulation with ischemia, and strangulation with necrosis (*P*<0.01). Compared with the PT, only the coagulation index was significantly different between the simple and necrosis subgroups (*P*<0.05). The Fib level was higher in the simple group relative to the strangulated group (both the ischemia subgroup and the necrosis subgroup) (*P*<0.05). This result implied that an elevated Fib level was not only due to acute-phase protein but also coagulation factors. Therefore, the Fib level can be used to indirectly reflect the degree of intestinal obstruction and intestinal necrosis as well as intestinal blood flow in the case of intestinal obstruction.

In the past, the WBC had been used as an indicator for the diagnosis of strangulation ileus, but our data showed that the WBC was not significantly different between the 2 groups. We compared WBC, CRP, and Fib by ROC curve analysis and found that the areas under the ROC curve for CRP (0.78) and Fib (0.80) were greater than that for WBC (0.58). Therefore, CRP and Fib have a greater value in the diagnosis of strangulated intestinal obstruction. As infection often occurs from simple intestinal obstruction to strangulated intestinal obstruction, our data also showed that inflammatory markers like CRP and NEUT also increased. In particular, the CRP level was significantly different between the simple intestinal obstruction group and the strangulated intestinal obstruction group (*P*<0.01), as well as the ischemic subgroup and the necrosis subgroup of the patients with strangulated intestinal obstruction (*P*<0.01). We confirmed that the CRP level was able to reflect the inflammatory condition of strangulated intestinal obstruction with necrosis, which was consistent with previous studies.^6^,^14^,^15^ In addition, the Fib level was positively correlated with the CRP level (*r*=0.63, *P*<0.01) but was negatively correlated with the TT (*r*=−0.54, *P*<0.01). Our results showed that coagulation indicators, except for indicators of inflammation, can also reflect intestinal strangulation of intestinal obstruction. Meanwhile, we demonstrated that an elevated level of Fib in strangulated intestinal obstruction was caused by coagulation factors. Moreover, our findings also showed that a combination of coagulation indicators and inflammatory markers may be better for the diagnosis of strangulated intestinal obstruction. Fib and CRP have high specificity and positive predictive value, indicating that both of them have a certain significance for intestinal strangulation in patients with intestinal obstruction (but other factors affecting coagulation must be excluded).

The value of lactic acid in intestinal strangulation was rarely reported. Because only a few patients in this group had examined lactic acid, it was not analyzed in our study.

## CONCLUSIONS

In summary, Fib and CRP showed a good ability to distinguish between strangulated intestinal obstruction and simple intestinal obstruction. Therefore, coagulation indicators and inflammatory markers have the potential to be used as markers for predicting strangulation in patients with intestinal obstruction.
